# Maximum growth and survival of estrogen receptor-alpha positive breast cancer cells requires the Sin3A transcriptional repressor

**DOI:** 10.1186/1476-4598-9-263

**Published:** 2010-09-29

**Authors:** Stephanie J Ellison-Zelski, Elaine T Alarid

**Affiliations:** 1 Department of Oncology, University of Wisconsin-Madison, Madison, Wisconsin 53706, USA

## Abstract

**Background:**

Sin3A is an evolutionarily conserved transcriptional repressor which regulates gene expression as part of the multi-protein Sin3 repressive complex. It functions as a scaffold upon which proteins with enzymatic activity dock, including chromatin modifying histone deacetylases. Although regulation of transcription by Sin3A has been studied in detail, little is understood about the function of Sin3A in cancer cells. We previously showed that Sin3A is expressed in breast cancer cells and is a repressor of estrogen receptor-alpha (ERα, *ESR1*) gene expression. Here, we expand our previous studies to elucidate the function of Sin3A in the control of gene expression and growth of breast cancer cells.

**Results:**

Analysis of gene expression following knockdown of Sin3A revealed changes in both basal and regulated gene transcription. Genes of known importance in breast cancer and estrogen signaling, including *ERBB2*, *PGR*, *MYC, CLU, *and *NCOA2, *were among those identified as Sin3A-responsive. The mechanism of Sin3A action varied among genes and was found to be mediated through both HDAC1/2 -dependent and -independent activities. Loss of Sin3A inhibited breast cancer cell growth by increasing apoptosis without affecting cell cycle progression. Analysis of both ERα-positive and ERα-negative cell lines revealed that the effects of Sin3A on growth were cell-type specific, as Sin3A expression promoted maximum growth of only the ERα-positive cells, and, notably, Sin3A protein itself was increased by estrogen. Further gene expression experiments revealed that Sin3A repressed expression of key apoptotic genes, including *TRAIL*, *TRAILR1*, *CASP10*, and *APAF1*, in ERα-positive, but not ERα-negative, cell lines, which could provide a mechanistic explanation for cell-type differences in growth.

**Conclusions:**

This study identifies Sin3A as a regulator of gene expression, survival, and growth in ERα-positive breast cancer cells. Sin3A regulates the transcription of genes involved in breast cancer and apoptosis and acts through multiple mechanisms not limited to histone deacetylase function. These findings reveal previously undescribed functions of Sin3A in breast cancer and provide evidence for an important role of this transcriptional repressor in ERα-positive tumor cell growth.

## Background

Appropriate regulation of genes is important in maintaining normal cell growth, and disruption of gene regulation is associated with human cancer. Changes in gene expression can distinguish types of breast tumors and predict response to therapies [1-3]. Tremendous effort, therefore, has been devoted to dissecting pathways that regulate transcription. For example, understanding the mechanisms of gene activation by estrogen receptor-alpha (ERα) was foundational in the development of hormonal therapy [4]. Interestingly, microarray analyses on estrogen-treated breast cancer cells show that the number of repressed genes is greater than or near the number of activated genes [5-8]. Although these experiments show that estrogen-mediated repression of genes is clearly biologically important, the mechanisms responsible for repression are not fully understood. We previously showed that the Sin3A transcriptional repressor protein is a regulator of estrogen-induced repression of the ERα gene, *ESR1*, in breast cancer cells [9]. Furthermore, it was found that Sin3A and ERα exist in an endogenous estrogen-responsive complex. These data suggested that Sin3A may play a broader role in ERα-positive breast cancer cells.

The role of Sin3A in breast cancer is virtually unexplored, but studies suggest that Sin3A is important in normal growth and may be a player in other neoplastic model systems. Homozygous deletion of Sin3A in mice is embryonic lethal, demonstrating that Sin3A serves essential developmental functions [10,11]. Studies using conditional Sin3A knockout in mouse embryonic fibroblasts (MEFs) find that Sin3A deletion leads to decreased proliferation and increased apoptosis of cells [10,11]. In cancer models, Sin3A function is less clear. Lymphoma and sarcoma cell lines derived from primary tumors arising in a p53^-/- ^background exhibit proliferative arrest and increased apoptosis upon Cre-mediated deletion of Sin3A, suggesting that Sin3A has oncogenic functions [11]. However, another report suggests that Sin3A functions as a tumor suppressor in non-small cell lung cancer (NSCLC), as down-regulation of Sin3A mRNA occurs in several cases of NSCLC [12]. These few reports with disparate findings highlight a fundamental lack of understanding of the role of Sin3A in growth and cancer.

At the molecular level, Sin3A functions as the scaffolding component of the multi-protein Sin3 repressor complex that mediates transcriptional repression of several genes. The Sin3 complex was identified in yeast but is conserved in species through mammals [13,14]. The characteristic catalytic activity associated with Sin3A is histone deacetylation via its interactions with HDAC1/2 [15,16]. Additional components of the complex consist of SAP18/30, which stabilize the Sin3A-HDAC interaction, and RbAp46/48, which anchor the Sin3 complex on nucleosomes [15-17]. Sin3A does not possess intrinsic DNA-binding activity, so it must be targeted via interaction with other DNA-bound factors. Interactions for numerous DNA-binding factors and Sin3A have been identified, including Mad, p53, MeCP2, NRSF, CTCF, and ERα as examples [9,18-22]. Sin3A can also interact with other enzymatic proteins, including those capable of histone methylation, DNA methylation, chromatin remodeling, and N-acetylglucoseamine transferase activity [20,23-28].

In this report, we expand our previous findings and identify the function of Sin3A in gene expression, survival, and growth of breast cancer cells. Gene expression analysis identified a specific subset of Sin3A-responsive genes that were regulated by both HDAC1/2-dependent and -independent mechanisms. Importantly, decreased Sin3A expression led to an increase in apoptosis and increased expression of several apoptotic genes, which translated into attenuation of cell growth of ERα-positive and not ERα-negative breast cancer cells. This study identifies Sin3A as an essential regulator of growth and survival of ERα-positive breast cancer cells, which may have important translational implications for breast cancer patients.

## Results

### Sin3A regulates basal expression and estrogen-induced responses of specific genes in breast cancer cells

**Figure 1:**
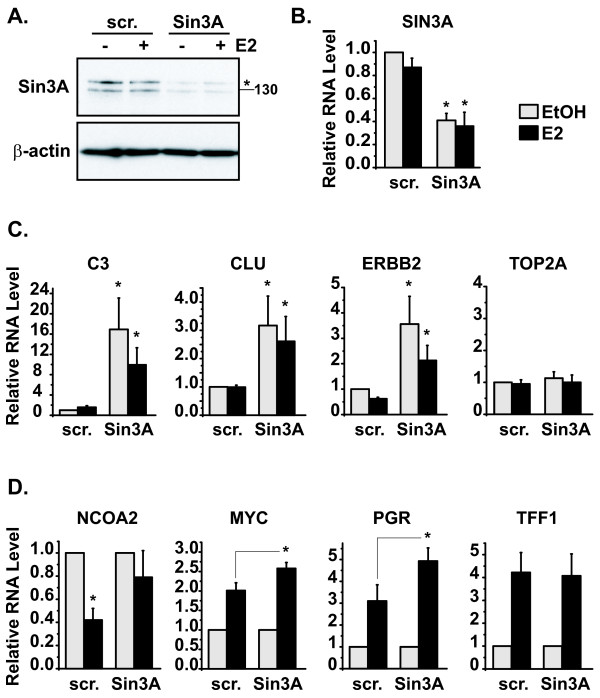
**Sin3A controls basal levels and estrogen-induced responses of genes in breast cancer cells**. MCF7 cells were transfected with scrambled (scr.) negative control or Sin3A siRNA, followed by treatment with vehicle control ethanol (EtOH) or 10 nM 17-β-estradiol (estrogen, E2) for four hours. **(A) **Knockdown of Sin3A protein was confirmed by western blot analysis. The band specific for Sin3A, which is a 145 kDa protein, is marked with an asterisk. The blot was reprobed with β-actin for the loading control. **(B) **Knockdown of *SIN3A *mRNA was also verified using quantitative reverse transcriptase real-time PCR (qRT-PCR), with ribosomal protein P0 serving as the housekeeping normalization gene. RNA levels were calculated relative to the scrambled ethanol sample. Data are from four independent experiments with error bars showing the standard error of the mean. Student's *t *tests were performed comparing corresponding scrambled and Sin3A siRNA samples, *p < 0.05. **(C) **qRT-PCR data, regulation of basal levels of genes, *C3*, *CLU*, *ERBB2*, and *TOP2A*. Experiments were performed and data calculated as above. **(D) **qRT-PCR data, regulation of estrogen responses of genes, *NCOA2*, *MYC*, *PGR*, and *TFF1*. Experiments were performed as above. RNA levels were calculated for each siRNA group relative to the corresponding ethanol-treated sample. Student's *t *tests were performed comparing estrogen responses in the presence of scrambled versus Sin3A siRNA for *MYC *and *PGR*, and determining significant repression of *NCOA2*, *p < 0.05.

Sin3A is a conserved multifunctional repressor protein present in organisms from yeast to mammals that functions by regulating gene transcription [17]. Our lab previously showed that Sin3A regulated the estrogen-induced repression of the ERα gene, *ESR1*, in the MCF7 breast cancer cell line [9]. To identify other genes regulated by Sin3A, MCF7 cells were transfected with a scrambled (scr.) negative control or Sin3A siRNA followed by treatment with vehicle ethanol (EtOH) or 10 nM 17-β-estradiol (estrogen, E2). Knockdown of Sin3A protein and mRNA by Sin3A siRNA were validated by western blot and quantitative reverse transcriptase real-time PCR (qRT-PCR), respectively (Figure 1A and 1B; the band for the Sin3A protein at the molecular weight of 145 kDa is marked with an asterisk). As an initial approach to identify Sin3A-regulated genes, a preliminary screen was performed of 84 genes that were identified based on their importance to breast cancer and estrogen signaling using a SABiosciences RT^2 ^Profiler PCR array. From this screen, 26 genes, including those that were and those that were not changed by Sin3A knockdown, were selected for validation by qRT-PCR in three independent experiments.

Expression data were analyzed for regulation by Sin3A, resulting in two groups of genes shown in Table 1: Sin3A responsive and Sin3A non-responsive. Sin3A responsive genes were those whose basal level (shown in bold) or estrogen response (shown in bold italic) were significantly (*p *< 0.05) changed in the presence of Sin3A siRNA compared to control scrambled transfected cells. For basal gene regulation by Sin3A, statistical analysis identified a 1.75 fold change as a cutoff for significant regulation. This resulted in eight Sin3A-responsive genes, all of which showed an increase in basal expression in the presence of Sin3A siRNA, in agreement with its role as a repressor of transcription. Graphs of qRT-PCR data from the three genes whose basal levels increased greatest with Sin3A knockdown, *C3*, *CLU*, and *ERBB2*, are shown in Figure 1C, along with an example of a gene whose levels did not change, *TOP2A*. For identifying genes whose estrogen responses were altered by Sin3A knockdown, any significant change in the response was allowed, resulting in four total genes. Sin3A siRNA prevented significant estrogen-induced repression of both *ESR1 *and *NCOA2*; *ESR1 *regulation by Sin3A had been shown previously by our lab [9]. The estrogen-induced activation of *MYC *and *PGR *were significantly increased in the presence of Sin3A siRNA, consistent with the loss of a transcriptional repressor. Graphs of qRT-PCR data from these genes are shown in Figure 1D, along with *TFF1 *whose estrogen response was not changed with Sin3A knockdown. The basal levels of the genes in Figure 1D did not change significantly (*NCOA2*, 1.34 ± 0.13; *MYC*, 1.24 ± 0.24; *PGR*, 0.88 ± 0.17; *TFF1*, 1.47 ± 0.35), and data are graphed as fold changes to highlight the magnitude of the estrogen response. Genes on the right-hand side of Table 1, Sin3A non-responsive, were those that did not statistically change either at the basal level or in the magnitude of their estrogen-response with Sin3A siRNA. These results identify a specific subset of genes regulated by Sin3A in breast cancer cells and show that Sin3A mediates both basal and regulated gene expression.

**Table 1:** Sin3A regulation of genes associated with breast cancer or estrogen receptor signaling

Sin3A Responsive	Sin3A Non-responsive
**C3**	CCND1
**CDH1**	CCNG2
**CDKN1A**	HMGB1
**CLU**	KRT18
**ERBB2**	KRT19
**GATA3**	MAP2K7
**MUC1**	NCOA3
**SERPINA3**	NME1
ESR1*	PIN1
***MYC***	SERPINB9
***NCOA2***	TFF1
***PGR***	TGFA
	TOP2A
	TP53

**bold **= significant (p < 0.05) increase in **basal level **of gene by Sin 3A siRNA

***bold italic ***= significant (p < 0.05) change in ***estrogen response ***of gene by Sin3A siRNA

* = previously identified [9]

### Effects of Sin3A on gene expression are mediated through HDAC1/2-dependent and-independent mechanisms

**Figure 2:**
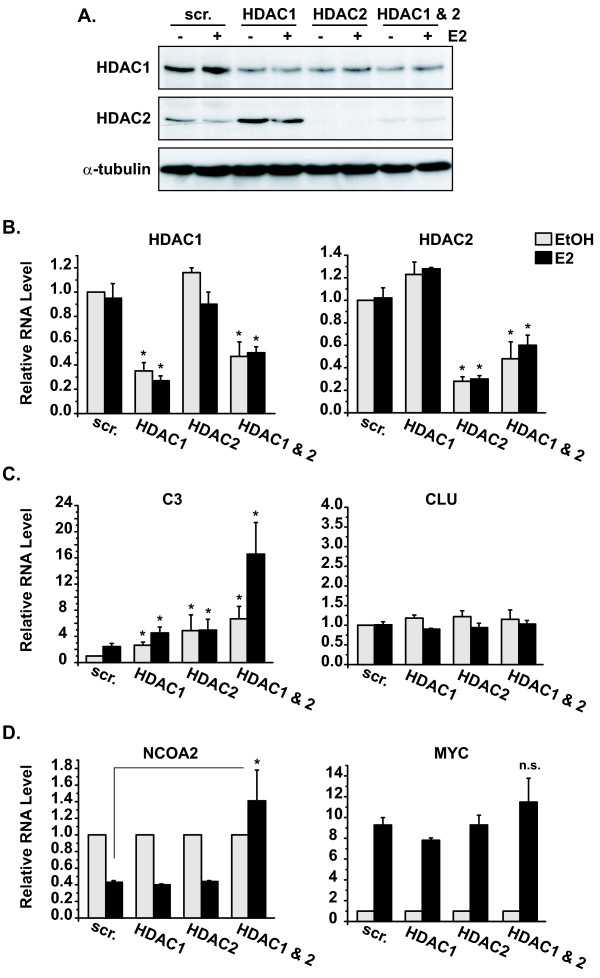
**Changes in gene expression by Sin3A are mediated through HDAC1/2-dependent and -independent pathways**. MCF7 cells were transfected with scrambled (scr.), HDAC1, HDAC2, or a combination of HDAC1 and HDAC2 siRNA, followed by treatment with ethanol (EtOH) or 10 nM estrogen (E2) for four hours. **(A) **Knockdown of HDAC1 and HDAC2 proteins was confirmed by western blot analysis, and the blot was reprobed with α-tubulin for the loading control. **(B) **Knockdown of HDAC1 and HDAC2 mRNA was also verified using qRT-PCR, with ribosomal protein P0 serving as the housekeeping normalization gene. RNA levels are calculated relative to the scrambled ethanol sample. Data are from three independent experiments with error bars showing the standard error of the mean. Student's *t *tests were performed comparing corresponding scrambled and HDAC1 or HDAC2 siRNA samples, *p < 0.05. **(C) **qRT-PCR data, regulation of basal levels of genes, *C3 *and *CLU*. Experiments were performed and data calculated as above. **(D) **qRT-PCR data, regulation of estrogen responses of genes, *NCOA2 *and *MYC*. Experiments were performed as above. RNA levels were calculated for each siRNA group relative to the corresponding ethanol-treated sample. Student's *t *tests were performed comparing estrogen responses in the presence of scrambled versus HDAC siRNAs, *p < 0.05. ns = not significant.

The enzymatic function characteristically associated with Sin3A is histone deacetylation via its core interactions with HDAC1 and HDAC2 [16]. However, several studies have shown that this function can be expanded by adding alternative catalytic components onto the Sin3A platform [20,23-28]. To gain mechanistic insight into Sin3A target gene regulation, siRNAs directed against HDAC1 and HDAC2 were used to establish whether changes observed in gene expression in Figure 1C and 1D were mediated via Sin3A's associated core histone deacetylase activity. MCF7 cells were transfected with scrambled negative control, HDAC1, or HDAC2 siRNA, followed by treatment with vehicle ethanol or estrogen. Experiments also included a double HDAC1 and HDAC2 knockout since both are present in the Sin3A complex. Knockdown of HDAC1 and HDAC2 protein and mRNA levels were verified by western blot and qRT-PCR analysis (Figure 2A and 2B). Although modest regulation of the proteins by the opposite siRNA was observed, the qRT-PCR data showed specific regulation at the transcript level by respective HDAC siRNAs.

Genes from Figure 1 (*C3, CLU, ERBB2, NCOA2, MYC, *and *PGR*) which were regulated by Sin3A were analyzed for changes in expression in the presence of decreased HDAC1 and HDAC2 levels. *C3*, whose basal levels increased in response to Sin3A siRNA (Figure 1C), also increased with the loss of HDAC1 or HDAC2 by siRNA (Figure 2C). In contrast, the levels of *CLU*, whose basal levels also increased in response to Sin3A siRNA, were not increased by any of the HDAC siRNAs, even in the double HDAC1 and HDAC2 sample (Figure 2C). *ERBB2 *showed similar results to *CLU *(data not shown). For regulated responses, the estrogen-induced repression of *NCOA2 *was reversed when both HDAC1 and HDAC2 were decreased (Figure 2D), similar to the reversal of repression observed with Sin3A siRNA (Figure 1D). Conversely, none of the HDAC siRNAs significantly affected the level of estrogen-induced activation of the *MYC *gene (Figure 2D) or *PGR *(data not shown). In sum, the loss of HDAC1 and HDAC2 increases *C3 *and *NCOA2*, but not *CLU*, *ERBB2*, *MYC*, or *PGR*, providing evidence that *C3 *and *NCOA2 *are repressed in breast cancer cells by the HDAC1/2 components of the Sin3A repressive complex. These data establish that changes mediated by Sin3A in both basal and estrogen responses of genes involve HDAC1/2-dependent and -independent mechanisms and are gene specific.

### Loss of Sin3A promotes apoptosis of breast cancer cells but does not affect cell cycle progression

Gene expression studies described above showed that loss of Sin3A affected a specific subset of genes involved in breast cancer by both increases in the basal level and modulation of estrogen responses. Furthermore, some mechanisms of regulation involved the HDAC1/2 activity of the core Sin3A complex (*C3, NCOA2*), while others involved alternative capabilities (*CLU, MYC*). Together, this suggested that Sin3A was a master scaffolding protein whose broad effects on genes may translate into an effect on cell growth. Few studies have been conducted on the role of Sin3A in growth of mammalian cells, and these few reports have suggested conflicting roles for Sin3A in cancer [11,12].

**Figure 3:**
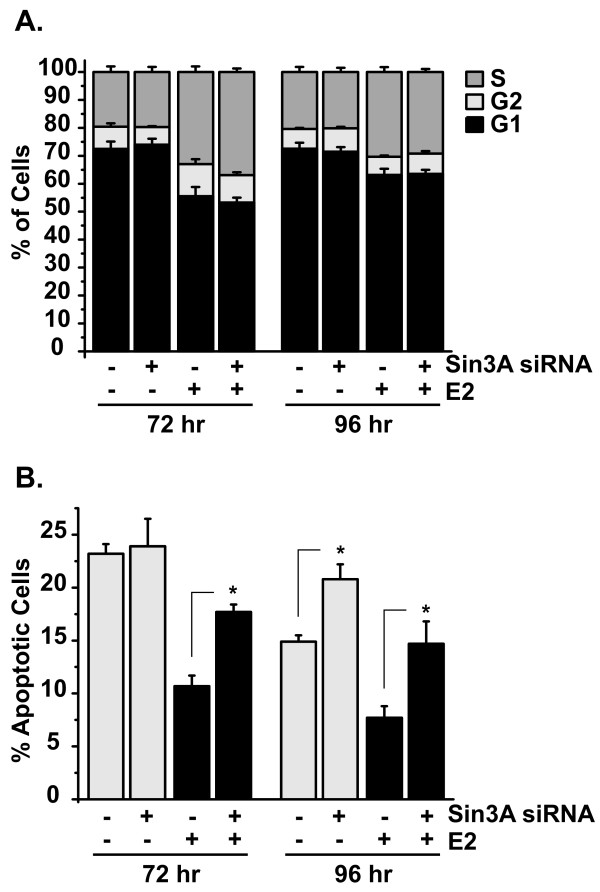
**Loss of Sin3A increases apoptosis but does not affect the cell cycle of breast cancer cells**. MCF7 cells were transfected with scrambled negative control or Sin3A siRNA and treated with vehicle ethanol or 10 nM estrogen (E2) for 72 or 96 hours. Samples were analyzed simultaneously by flow cytometry for **(A) **cell cycle distribution and **(B) **annexin V, indicative of apoptotic cells. Data are from three independent experiments with error bars showing the standard error of the mean. To determine statistical significance of the findings in (B), student's *t *tests were performed comparing corresponding scrambled and Sin3A siRNA samples, *p < 0.05.

**Figure 4:**
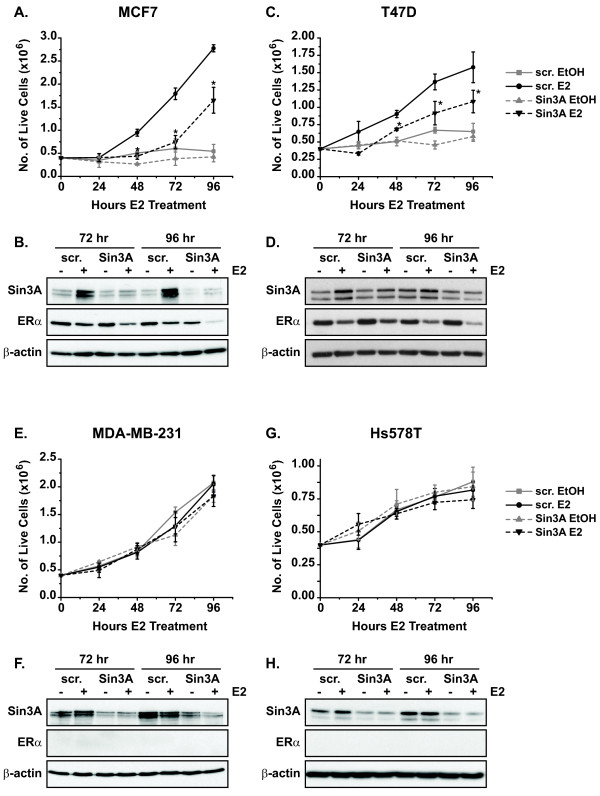
**Sin3A is required for maximum growth of ERα-positive, but not ERα-negative, breast cancer cells**. ERα-positive **(A, B) **MCF7 and **(C, D) **T47D cells, or ERα-negative **(E, F) **MDA-MB-231 and **(G, H) **Hs578T cells, were transfected with either scrambled (scr.) negative control or Sin3A siRNA. Cells were then treated with vehicle ethanol (EtOH) or 10 nM estrogen (E2). Cells from each group were harvested at daily intervals, and the number of live cells was determined by trypan blue exclusion and counting. Error bars show the standard error of the mean of three independent experiments. Lysates from 72 and 96 hour time points were subjected to western blot analysis to confirm efficient knockdown of Sin3A (B, D, F, H). Blots were reprobed for ERα and the loading control, β-actin. To determine significance of loss of Sin3A on estrogen-induced growth of cells in (A) and (C), student's *t *tests were performed comparing scrambled and Sin3A siRNA samples at each time point, *p < 0.05.

Flow cytometry analysis was performed on Sin3A knockdown cells to determine the role of Sin3A in cell cycle progression of breast cancer cells. MCF7 cells were transfected with scrambled or Sin3A siRNA and treated with or without estrogen for 72 or 96 hours. At 72 hours, there was no difference in the cell cycle distribution, determined by Hoescht dye intensity, of scrambled and Sin3A siRNA cells treated with vehicle ethanol (Figure 3A). Similarly, there was no difference in the cell cycle distribution of cells transfected with scrambled or Sin3A siRNA and treated with estrogen. As expected, estrogen treatment resulted in an increase in the percentage of cells in the S/G2/M phases and a subsequent decrease in G0/G1 phases [29]. Similar results were found with the 96 hour samples, shown in the right panel of Figure 3A, indicating that Sin3A is not involved in cell cycle progression of MCF7 cells. Knockdown of Sin3A under these conditions was verified and is shown in Figure 4B.

The samples described above were analyzed simultaneously for Annexin V staining to assess the level of cellular apoptosis. At the 72 hour time point, there was no difference in apoptosis of scrambled and Sin3A siRNA transfected cells treated with vehicle ethanol (Figure 3B). Estrogen treatment in control transfected cells decreased the level of apoptosis compared to ethanol (first bar versus third bar in Figure 3B). However, this level of apoptosis was significantly increased with loss of Sin3A (third bar versus fourth bar in Figure 3B). By 96 hours, there was a significant increase in apoptosis in Sin3A siRNA knockdown cells both in the presence and absence of estrogen. This identifies Sin3A as a prosurvival factor in MCF7 cells that protects against apoptosis.

### Sin3A is required for maximum growth of ERα-positive breast cancer cells

Cell growth assays were performed to determine if the observed increase in apoptosis by Sin3A knockdown was sufficient to attenuate growth of breast cancer cells. Since the increase in apoptosis at 96 hours occurred both in the presence and absence of estrogen (Figure 3B), the growth of ERα-negative (MDA-MB-231 and Hs578T) as well as ERα-positive (MCF7 and T47D) cell lines was analyzed. Cells were transfected with either the negative control scrambled or Sin3A siRNA, treated with vehicle ethanol or estrogen, and the number of live cells was counted every 24 hours by trypan blue exclusion (Figure 4). MCF7 cells transfected with the scrambled negative control grew steadily in the presence of estrogen, but no cell growth was observed in the scrambled ethanol group, consistent with the fact that this is an estrogen-dependent cell line (Figure 4A). Notably, a significant decrease in estrogen-induced growth of MCF7 cells was exhibited by those transfected with Sin3A siRNA. MCF7 cells transfected with the Sin3A siRNA but treated with ethanol also tended to have lower cell numbers than the corresponding scrambled control, but data were not statistically significant. T47D cells, another ERα-positive cell line, also exhibited significant attenuation of estrogen-induced growth in the presence of Sin3A siRNA (Figure 4C). The growth defect was not as dramatic as that observed in MCF7 cells. It is of note that knockdown of Sin3A protein, shown by Western blot analysis in Figure 4D, was also less efficient in the T47D cells. In contrast to the ERα-positive cell lines, ERα-negative MDA-MB-231 and Hs578T cells grew at the same rate in the presence and absence of Sin3A siRNA or estrogen (Figure 4E and 4G). These data provide evidence that Sin3A is required for maximum growth of ERα-positive MCF7 and T47D cells but not ERα-negative MDA-MB-231 and Hs578T cells.

**Figure 5:**
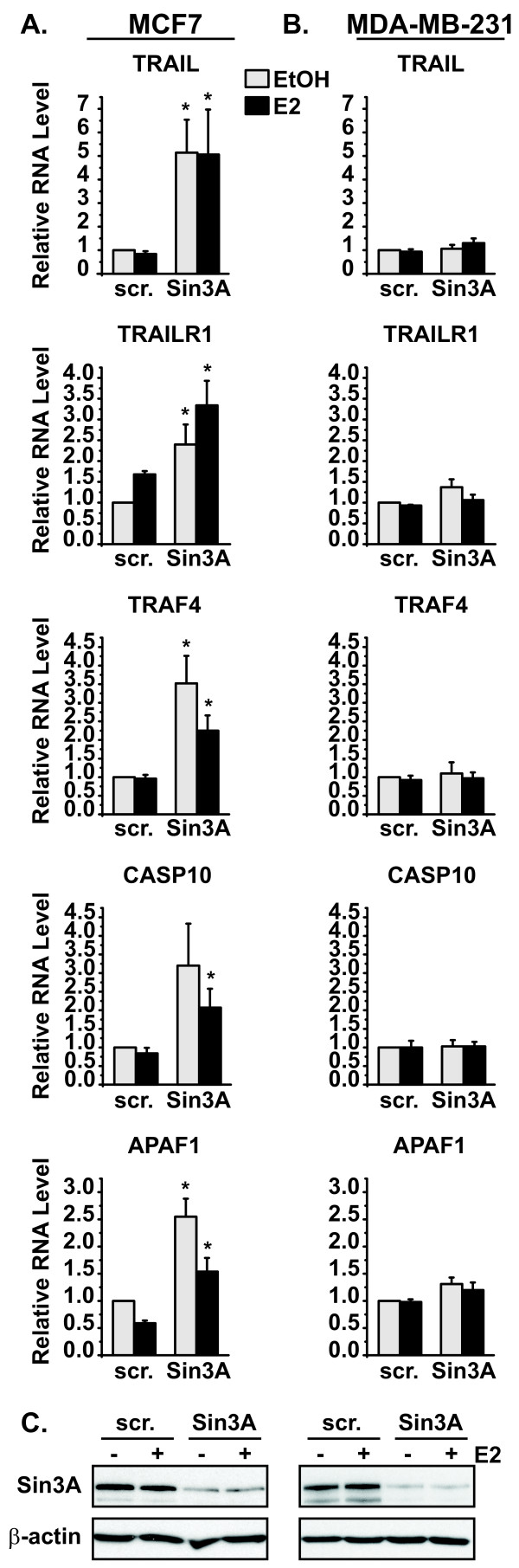
**Loss of Sin3A increases expression of apoptotic genes differentially in ERα-positive and ERα-negative cell lines**. **(A) **MCF7 or **(B) **MDA-MB-231 cells were transfected with scrambled (scr.) negative control or Sin3A siRNA, followed by treatment with vehicle control ethanol (EtOH) or 10 nM estrogen (E2) for four hours. qRT-PCR was performed for the indicated apoptotic gene, with ribosomal protein P0 serving as the housekeeping normalization gene. RNA levels were calculated relative to the scrambled ethanol sample. Data are from a minimum of three independent experiments with error bars showing the standard error of the mean. Student's *t *tests were performed comparing corresponding scrambled and Sin3A siRNA samples, *p < 0.05. **(C) **Knockdown of Sin3A protein was also confirmed by western blot analysis in each cell line. The blot was reprobed with β-actin for the loading control.

Western blot analyses in Figure 4B, D, F, and 4H confirmed that Sin3A was decreased and could not account for different dependencies on Sin3A for growth in ERα-positive versus ERα-negative cell lines. Interestingly, western blot analysis revealed a robust estrogen-induced increase in Sin3A protein levels in control transfected MCF7 cells at both 72 and 96 hours (Figure 4B). This increase was also observed in T47D cells, albeit to a lesser extent (Figure 4D). Data from earlier time points of four hours estrogen treatment did not show this increase in Sin3A, suggesting that it is a long-term or secondary response (Figure 1A and 5C). Further estrogen time course western blot experiments showed that Sin3A protein levels increased by 24 hours of estrogen treatment in MCF7 cells, and this was sustained at a similar level out to 96 hours (Additional File 1A). The increase in Sin3A protein was independent of effects on transcription of *SIN3A *mRNA (Additional File 1B). Estrogen treatment did not affect the levels of *SIN3A *mRNA at any time point, but estrogen did decrease the levels of *ESR1 *as a positive control for estrogen responsiveness (Additional File 1C) [9,30]. The observation that high levels of Sin3A protein are maintained with long-term estrogen treatment further supports its role in promoting survival of ERα-positive cells.

### Sin3A differentially represses expression of key apoptotic genes in ERα-positive versus ERα-negative breast cancer cells

Data in Figure 3 suggested that Sin3A affected growth of ERα-positive cells by regulation of apoptosis and not cellular proliferation. To provide mechanistic insight into the increase in apoptosis, qRT-PCR analysis was performed to identify Sin3A-regulated apoptotic genes. MCF7 cells were transfected with scrambled or Sin3A siRNA and treated with ethanol or estrogen, as in Figure 1. Several apoptotic genes were analyzed, including those involved in both the extrinsic death receptor and the intrinsic mitochondrial stress signaling pathways [31]. Genes involved in the extrinsic death receptor pathway that were significantly increased upon loss of Sin3A in MCF7 cells were the apoptotic-inducing ligand *TRAIL *(also known as *TNFSF10 *or *APO2L*), one of its receptors *TRAILR1 *(also termed *TNFRSF10A*, *APO2*, or *DR4*), mediators *TRAF4 *and *TRADD*, and *CASP10 *(Figure 5A and Additional File 2A). Other genes implicated in extrinsic death signaling that were tested but not significantly altered by Sin3A knockdown were *TNF *ligand, death receptors *TNFRSF25 *(also called *APO3 *or *DR3*) and *FAS*, and the *FADD *mediator (data not shown). Three genes involved in the intrinsic mitochondrial stress signaling apoptotic pathway were also significantly increased with loss of Sin3A in MCF7 cells - *APAF1*, *BNIP3L*, and *CASP9 *(Figure 5A and Additional File 2A). Notably, *CASP9 *was repressed by estrogen treatment, and this repression was reversed in the presence of Sin3A siRNA (Additional File 2A). Expression of another key mitochondrial stress gene, *BCL2*, was not responsive to Sin3A (data not shown).

Regulation of apoptotic genes was also determined in ER-negative MDA-MB-231 cells. qRT-PCR analysis was performed on genes from cells transfected with Sin3A siRNA and found that the majority of apoptotic genes were repressed by Sin3A in MCF7 cells, but not MDA-MB-231 cells (Figure 5B and Additional File 2B). Specifically, *TRAIL*, *TRAILR1*, *TRAF4*, *CASP10*, and *APAF1 *were not significantly altered by loss of Sin3A in MDA-MB-231 cells (Figure 5B). Three genes, *TRADD*, *BNIP3L*, and *CASP9*, were significantly increased in MDA-MB-231 cells transfected with Sin3A siRNA, but to a lower level than MCF7 cells (Additional File 2B). Western blot analysis in Figure 5C again confirmed that Sin3A levels were efficiently decreased in both cell lines and could not be the reason for the disparate gene regulation. Together, these data show that Sin3A differentially regulates the expression of apoptotic genes in ERα-positive MCF7 cells and ERα-negative MDA-MB-231 cells, which may selectively influence the growth and survival of the ERα-positive subtype of breast cancer.

## Discussion

Previous studies had suggested a role for Sin3A in growth of normal and neoplastic cells, but the function of Sin3A in breast cancer had not been fully explored [10,11]. Prior research from our lab identified Sin3A as a regulator of ERα gene, *ESR1*, expression and found an estrogen-responsive interaction between ERα and Sin3A [9]. This led us to further determine Sin3A regulation of gene expression and growth in breast cancer cells. We find that Sin3A regulates a subset of genes in ERα-positive MCF7 cells through both HDAC1/2-dependent and -independent activities. Maximum growth and survival of ERα-positive MCF7 and T47D cells requires expression of Sin3A. Interestingly, we also find that estrogen causes an increase in Sin3A protein levels in ERα-positive cells, suggesting the involvement of Sin3A in a feedback circuit regulating estrogen-dependent growth of breast cancer cells. Further, Sin3A represses important apoptotic genes in ERα-positive cell lines, consistent with our finding that decreased Sin3A levels leads to cellular apoptosis.

This study identifies the transcriptional repressor, Sin3A, as a necessary survival factor in ERα-positive breast cancer cells. Our data further support the idea that Sin3A promotes growth and survival of cells proposed in previous studies [10,11]. Together, these results raise the intriguing possibility that gene repression is as important of a determinant for cell growth as gene activation, as Sin3A primarily functions as a repressor. Other chromatin modifying repressor proteins, including the MTA components of the Mi-2/NuRD complex and EZH2, are also associated with breast cancer growth and progression [32-35]. Our identification of Sin3A as a prosurvival factor is further interesting in that it highlights the importance of estrogen-mediated survival of breast cancer cells. Sin3A knockdown increased apoptosis but had no effect on the cell cycle (Figure 3). Estrogen is commonly viewed as a mitogenic agent that increases growth of breast cancer cells through cell cycle progression, but our data support the notion that estrogen-mediated repression of apoptosis also has a large impact on overall growth of cells [29,36].

Clinical trials for HDAC inhibitors in breast cancer treatment, such as vorinostat, are still in early phases and often involve patients with advanced disease [37-40]. These studies have seen only partial efficacy that often increases when in combination with other agents, such as chemotherapy, and there are often issues of toxicity [37-40]. Our data suggest that developing therapeutic agents to target the scaffolding component of HDAC-complexes, such as Sin3A, may be of value, particularly because Sin3A affected only a subset of genes, but its loss still caused cell death. An agent that could disrupt Sin3A would target both its HDAC-dependent and -independent activities, possibly expanding the efficacy beyond that of HDAC inhibitors. Other reports support the finding that Sin3A has both HDAC1/2-dependent and -independent capabilities. For example, in stem cells, Sin3A is the key member of the Sin3 complex involved in the regulation of *NANOG *gene expression, not HDAC1/2 [41]. Several studies have also shown that Sin3A can interact with histone methylases (Smyd2, Set1/Ash2, and ESET), DNA methylation proteins (MeCP2), chromatin remodeling enzymes (ISWI, Brg1, hBrm, and BAF155), and O-linked N-acetylglucosamine transferase (OGT), demonstrating that Sin3A has the potential to serve as an integrator of broad transcriptional and epigenetic changes in cells [20,23-28]. Furthermore, *in vitro *transcription reactions on reconstituted nucleosomal templates find that addition of the HDAC inhibitor, trichostatin A (TSA), abolishes Sin3A-mediated repression of an acetylated histone H3 template, but not acetylated histone H4 template [42]. This *in vitro *experiment shows that Sin3A, even in the absence of other repressor molecules and enzymatic proteins, possesses some intrinsic HDAC1/2-independent capabilities.

Our data show that loss of Sin3A increases apoptosis of ERα-positive MCF7 cells (Figure 3). Upon further mechanistic experiments, we find that several genes with known roles in apoptosis are increased with Sin3A knockdown. This suggests that Sin3A normally represses their expression in MCF7 cells to aide in preventing apoptosis, and subsequently, promote cell growth. The apoptotic gene targets we identified fall into both the extrinsic death receptor and intrinsic mitochondrial apoptotic signaling pathways [31]. Interestingly, we find that Sin3A regulates genes involved in all steps of the extrinsic pathway in MCF7 cells - ligands, death receptors, adaptors, and caspases (Figure 5A and Additional File 2A) [43]. Specifically, levels of the *TRAIL *ligand, and its receptor, *TRAILR1*, are increased in MCF7 cells with Sin3A siRNA. *TRAIL *is a member of the tumor necrosis factor (TNF) superfamily of cytokines which can induce apoptosis by binding to extracellular domains of one of its receptors, which includes *TRAILR1*, a member of the TNF receptor superfamily [44-46]. Death receptors further use interactions with intracellular adaptor proteins to mediate signals from the extracellular environment, and loss of Sin3A increased levels of both *TRADD *and *TRAF4 *adaptors [47,48]. Lastly, we identify *CASP10 *as a Sin3A-responsive gene. In addition to caspase 8, caspase 10 has been shown to act as an initiator caspase in the death receptor signaling pathway, which can lead to activation of downstream executioner caspases to cause apoptosis [49].

Three genes connected to the intrinsic mitochondrial apoptotic-inducing pathway are also regulated by Sin3A in MCF7 cells - *APAF1*, *CASP9*, and *BNIP3L *(Figure 5A and Additional File 2A). The involvement and connection of Apaf-1 and caspase 9 in stress-induced apoptosis has been studied in great detail. Briefly, cellular stress stimulates release of cytochrome c from the mitochondria where it can then bind to Apaf-1, inducing conformational changes, ATP hydrolysis, and multimerization of Apaf-1 [50]. The complex, referred to as the "apoptosome", then recruits and activates procaspase 9, and active caspase 9 can cleave executioner caspases to cause apoptosis [51,52]. Finally, *BNIP3L *(also known as *NIX*) is a proapoptotic member of the Bcl-2 family of proteins that function upstream of the apoptosome to regulate the release of cytochrome c from the mitochondria [53,54]. Our findings that Sin3A regulates key genes from both the death receptor and mitochondrial stress apoptotic-inducing pathways emphasize the importance of this transcriptional repressor.

Our data find that Sin3A differentially regulates the expression of the apoptotic genes discussed above in ERα-positive (MCF7) and ERα-negative (MDA-MB-231) cell lines. *TRAIL*, *TRAILR1*, *TRAF4*, *CASP10*, and *APAF1 *increase upon Sin3A knockdown in MCF7, but not MDA-MB-231, breast cancer cells (Figure 5). Three genes, *TRADD*, *BNIP3L*, and *CASP9*, increase in both cell lines with loss of Sin3A, demonstrating that Sin3A possesses some overlapping gene regulation between breast cancer cell lines, as may be expected (Additional File 2). However, it is of note that the increase seen in these three genes upon Sin3A knockdown is greater in the MCF7 cells. Differences in apoptotic gene regulation by Sin3A in ERα-subtypes can mechanistically explain the discrepancies seen in effects of Sin3A on cell growth. Induction of apoptotic genes in the ERα-positive MCF7, and not ERα-negative MDA-MB-231 cells, could lead to increases in apoptosis and a resulting decrease in cell growth, as we observe. Furthermore, Sin3A protein itself is increased by estrogen in the ERα-positive breast cancer cell lines, discussed below.

To our knowledge, this is one of the first studies to identify a regulator of Sin3A levels - estrogen. Most studies concerning Sin3A have focused on its ability to regulate expression of other genes, and little knowledge exists about how levels of Sin3A itself are modulated. Another study has shown that Sin3A can be sumoylated by TOPORS, but other modulators of Sin3A are virtually unknown [55]. We observe an estrogen-induced increase in Sin3A protein levels that occurs independent of effects on Sin3A mRNA, demonstrating that regulation of Sin3A occurs via nongenomic actions (Additional File 1). This suggests that differences in Sin3A expression would not be found in microarray studies, possibly explaining why the role of Sin3A in breast cancer has not been appreciated until now. The mechanism by which estrogen treatment increases Sin3A protein levels is likely via a secondary effect since elevated levels are not seen before 24 hours (Figure 1A and 5C). Different ERα-positive cell types also seem to have a greater dependency on Sin3A levels for survival and growth than others. For example, we find a greater induction of Sin3A protein in MCF7 than T47D cells, and subsequently, a greater effect of Sin3A on growth of MCF7 cells (Figure 4).

While this manuscript was under revision, another group published that the Sin3 complex represses the ERα gene, *ESR1*, in ERα-negative breast cancer cell lines [56]. Consistent with this finding, we showed Sin3A regulation of estrogen-induced repression of *ESR1 *in MCF7 cells [9]. However, unlike the other publication, we did not observe reexpression of either *ESR1 *mRNA (data not shown) or ERα protein (Figure 4F) in MDA-MB-231 in our current studies. These discrepancies may be due to different experimental conditions and techniques. Importantly, the authors in [56] disrupted Sin3A and Sin3B function by using the Sin3 interaction domain (SID) of the MAD protein, while our experiments focused only on Sin3A. Additionally, the SID from MAD may participate in other protein interactions beyond the Sin3 proteins. Together, these reports suggest that components besides Sin3A are necessary to mediate the repression of *ESR1 *in ERα-negative cells.

Finally, our data show several converging points between Sin3A and the estrogen signaling pathway. As described above, estrogen increases protein levels of Sin3A, suggesting a feedback loop to control estrogen-dependent growth. Our previous report shows that Sin3A controls expression of the ERα gene itself, *ESR1*, and Sin3A can interact with ERα in ERα-positive breast cancer cells [9]. We further show here that Sin3A controls expression of *NCOA2, *a member of the p160 coactivator family involved in mediating ERα transcriptional activation (Figure 1D) [57]. The estrogen-induced activation of *PGR*, which encodes the progesterone receptor (PR), also increases upon Sin3A knockdown (Figure 1D). PR status is often used as a marker of estrogen sensitivity and predictor of response to endocrine therapy in breast cancer [58,59]. Additionally, knockdown of Sin3A only prevents growth of ERα-positive MCF7 and T47D cells, not ERα-negative MDA-MB-231 and Hs578T cells, further supporting the notion that components intrinsic to the ERα signaling pathway are involved in mediating the ability of Sin3A to promote survival.

## Conclusions

This is one of the first studies to analyze the role of the Sin3A transcriptional repressor protein in breast cancer. We find that Sin3A regulates the expression of several genes important in breast cancer and estrogen signaling, and these effects are mediated through both HDAC1/2-dependent and -independent mechanisms of Sin3A. Our findings show that Sin3A is a prosurvival protein that promotes growth of ERα-positive breast cancer cells by preventing apoptosis through repression of key proapoptotic genes. These findings suggest that Sin3A may be a new therapeutic target, and identification of an agent that could disrupt Sin3A may be effective in controlling survival of ERα-positive tumors.

## Methods

### Cell Culture and Hormone Treatments

MCF7, MDA-MB-231, and Hs578T cells were maintained at 37°C and 10% CO_2 _in Dulbecco's modified Eagle's medium (DMEM; Mediatech, Inc., Manassas, VA, USA) with phenol red and L-glutamine, supplemented with 10% fetal bovine serum (FBS; Biowest, Miami, FL, USA), 100 units/ml penicillin, and 100 μg/ml streptomycin (GIBCO/Invitrogen, Carlsbad, CA, USA). T47D cells were maintained at 37°C and 5% CO_2 _in RPMI 1640 medium with phenol red and L-glutamine (GIBCO), supplemented with 10% FBS, penicillin, and streptomycin as above. For hormone treatments, all cell lines were incubated at 37°C and 5% CO_2 _for at least three days in the media described above but without phenol red and containing six-times charcoal dextran stripped FBS. 17-β-estradiol (estrogen, E2; Steraloids, Inc., Newport, RI, USA) was added to a final concentration of 10 nM in all experiments for the length of time indicated in the figures. Ethanol (EtOH) vehicle control was 0.1% in all samples.

### Transfection of siRNA

One day prior to transfection, cells were plated in 10 cm plates at a density of 2 × 10^6 ^cells in antibiotic free media. 800 pmol of siRNA was diluted in Lipofectamine reagent (Invitrogen) and Opti-MEM (GIBCO) and added to appropriate plates for five hours. Three days later, cells were transfected with siRNA again as above in order to achieve maximum silencing. siRNA duplexes for Sin3A, HDAC1, HDAC2, and a scrambled negative control were predesigned and purchased from Sigma (Saint Louis, MO, USA).

### RNA Isolation and Quantitative RT-PCR

RNA isolation and quantitative reverse transcriptase real-time PCR (qRT-PCR) were carried out as previously detailed [9]. Primer sequences are available upon request. Ribosomal protein P0 mRNA was used as the internal control. Relative mRNA levels were calculated using the ΔΔCt method [60]. For initial screening of candidate Sin3A-regulated genes, two complimentary trial RT^2 ^Profiler Human Breast Cancer and Estrogen Receptor Signaling PCR arrays were used (SABiosciences now a Qiagen company, Frederick, MD, USA).

### Western Blot Analysis

Western blot analysis was carried out as previously described [61]. The primary antibodies used in this study were Sin3A (K-20, sc-994, Santa Cruz Biotechnology, Santa Cruz, CA, USA), ERα (VP-E613, Vector Laboratories, Burlingame, CA, USA), HDAC1 (H-11, sc-8410, Santa Cruz), HDAC2 (C-8, sc-9959, Santa Cruz), β-actin (A5441, Sigma), and α-tubulin (DM1A, CP06, Calbiochem, San Diego, CA, USA).

### Cell Growth Assays

Cells were transfected with scrambled or Sin3A siRNA as detailed above, changing the media to phenol-red free media the day before the second transfection. The day after the second transfection, cells were harvested and plated in 6-well plates at a density of 4 × 10^5 ^live cells, as determined by trypan blue exclusion and counting on a hemacytometer. Cells were then treated with either 10 nM E2 or EtOH. At 24 hour intervals, cells were harvested and resuspended in media. The number of live cells at each time point was determined by hemacytometer counting and trypan blue exclusion, taking the average of two counts for each sample in each experiment.

### Flow Cytometry for Cell Cycle and Apoptosis Analysis

Knockdown of Sin3A and hormone treatments were performed as described above. 72 and 96 hours post-treatment, media and cells were harvested and diluted to 1 × 10^5 ^cells in 1 ml of media. Hoescht (Invitrogen) was added at 10 μg/ml to the cells, and samples were incubated for 30 minutes at 37°C. Cells were spun down and resuspended in 100 μl of annexin binding buffer (10 mM HEPES, 140 mM NaCl, and 2.5 mM CaCl_2_, pH 7.4) containing Hoescht. 5 μl of the annexin V- Alexa Fluor 647 conjugate (Invitrogen) was added to the cell suspension and incubated for 15 minutes at room temperature. After the incubation, an additional 400 μl of annexin binding buffer was added, followed by propidium iodide (Sigma) to a concentration of 5 μg/ml. Dye intensities of 10,000 events were measured on the LSRII machine from BD Biosciences (San Jose, CA, USA) equipped with a UV laser. Apoptosis levels were analyzed using FlowJo software (Tree Star, Inc., Ashland, OR, USA), and cell cycle data were analyzed using ModFit software (Verity Software House, Topsham, ME, USA).

### Statistical Analysis

Error bars in all figures are the standard error of the mean of a minimum of three independent experiments. Statistics were performed using OrginLab (OrginLab Corp., Northampton, MA, USA), with the exact test described in the corresponding figure legend. Data were considered significant if *p *< 0.05 and are indicated in the figures by asterisks.

## Competing interests

The authors declare that they have no competing interests.

## Authors' contributions

SE designed and performed experiments, analyzed data, and wrote the manuscript. EA assisted with experimental design, data analysis, and writing of the manuscript. All authors read and approved the final manuscript.

## Supplementary Material

Additional file 1 **Estrogen regulates protein levels of Sin3A in ERα-positive breast cancer cells**. **(A) **MCF7 cells were treated with ethanol for 96 hours or 10 nM estrogen (E2) for the indicated length of time. Cell lysates were analyzed for Sin3A and β-actin protein expression by western blot. **(B, C) **MCF7 cells were treated as in (A), and RNA was isolated and reverse transcribed. Quantitative reverse transcriptase real-time PCR (qRT-PCR) was performed to determine the expression pattern of (B) *SIN3A *and (C) *ESR1*. Data are calculated relative to the ethanol-treated control, using ribosomal protein P0 as the housekeeping gene for normalization. Error bars show the standard error of the mean of three independent experiments. Click here for file

Additional file 2 **Additional apoptotic genes regulated by Sin3A in breast cancer cell lines**. **(A) **MCF7 or **(B) **MDA-MB-231 cells were transfected with scrambled (scr.) negative control or Sin3A siRNA, followed by treatment with vehicle control ethanol (EtOH) or 10 nM estrogen (E2) for four hours. qRT-PCR was performed for the indicated apoptotic gene, with ribosomal protein P0 serving as the housekeeping normalization gene. RNA levels were calculated relative to the scrambled ethanol sample. Data are from a minimum of three independent experiments with error bars showing the standard error of the mean. Student's *t *tests were performed comparing corresponding scrambled and Sin3A siRNA samples, *p < 0.05. Click here for file
